# Neural and Neurobehavioral Dysfunction in Children with Obstructive Sleep Apnea

**DOI:** 10.1371/journal.pmed.0030323

**Published:** 2006-08-22

**Authors:** Dean W Beebe

## Abstract

Beebe discusses a new study that found that children with obstructive sleep apnea scored significantly lower than controls on tests of overall intelligence and executive function.

Research has confirmed what many parents have long suspected: inadequate sleep in children is associated with daytime behavioral problems and poor academic functioning [1–3]. A fact less well-recognized by clinicians and the general public is that sleep-related breathing disorders, which occur in two to ten percent of children depending on how they are defined, can have a significant impact even among children who have normal sleep duration [4]. Snoring is a hallmark symptom of sleep-disordered breathing, and enlarged tonsils are often a contributing factor to the breathing disturbance. We still do not fully understand the mechanism that links snoring or enlarged tonsils with behavioral problems and poor grades.

One of the more severe nocturnal breathing disorders, obstructive sleep apnea (OSA), involves partial or complete breathing blockage recurrently during sleep, resulting in intermittent drops in blood oxygen levels and probable sleep disruption. Although brain processes are presumed to mediate the link between these medical factors and daytime behavioral and scholastic problems, our understanding of these processes remains fairly theoretical and speculative. The frontal and hippocampal regions of the brain, which are implicated in the regulation of behavior and memory, respectively, appear to be most vulnerable to OSA [4], but the evidence is indirect.

In a new study published in *PLoS Medicine*, Ann Halbower and colleagues [5] used proton magnetic resonance spectroscopy (MRS), a non-invasive neuroimaging technique that can detect chemical metabolites linked to neural dysfunction, to shed more direct light on the neural functioning of children with OSA.

## The Study Findings

Halbower and colleagues compared two groups of children aged six to 16 years. The first group was comprised of 19 children who were referred by clinicians to a sleep medicine program and found to have moderate to severe untreated OSA. The control group was comprised of 12 non-snoring children who were recruited from community advertisements and who were of similar age, ethnicity, sex, and household income to the group with OSA. All participants underwent standardized evaluations of their thinking skills, and most also underwent MRS.

Inadequate sleep in children is associated with daytime behavioral problems and poor academic functioning.

Compared with the controls, children with OSA scored significantly lower on tests of overall intelligence and some aspects of higher-level thinking called “executive functions,” but the groups did not differ on tests of sustained attention, motor skills, or visuospatial skills. Tests of memory did not yield significant differences between the groups, but the effect sizes were large enough for the authors to suggest that significant effects might have been found in a larger sample. MRS indicated that those with OSA had abnormal metabolites in the left hippocampus and right frontal cortex.

## Implications for Brain Development and Clinical Practice

These parallel findings of deficits on measures of behavioral and brain functioning in children with OSA are sobering, and lend support to concerns that OSA, if left untreated, can have substantial long-term adverse effects [4,6,7]. The developing brain does not simply unfold in a predetermined genetic process. Rather, it builds upon itself at each stage, with development dictated by the interaction of genes with the immediate cellular environment [8]. That environment is determined by the child's life experiences (e.g., reactions to OSA-related behavioral disturbances) and physiological functioning (e.g., OSA-related oxygen deprivation or sleep disruption). For this reason, untreated childhood OSA may have a particularly marked long-term impact.

If Halbower and colleagues' findings hold in follow-up research, pediatricians and other health-care professionals will need to become much better at consistently screening for symptoms of OSA. Sleep is rarely addressed in most pediatric clinics [9,10], even though simple clinical screening tools have been developed and tested [10]. This lack of clinical attention runs contrary to current evidence from sleep medicine and developmental neuroscience, which suggests that early disease detection and treatment should be a high priority.

## Limitations of the Study

There is reason to expect that Halbower and colleagues' findings will withstand replication, because they are consistent with adult studies that have shown similar abnormalities using MRS [11–13], and with prevailing theories of the mechanisms behind the daytime deficits observed in individuals with OSA. Still, further research is needed to verify and build on these findings. MRS provides indirect indices of neural dysfunction (not necessarily neuron death), and it is not clear whether those indices will normalize with effective OSA treatment or what long-term effects might persist. Similarly, although the current findings provided tantalizing suggestions of developmental effects, few children under age ten were able to tolerate the sedative-free MRS procedure, which requires lying still during the scan. As a result, the high-risk period for OSA in preschool and early grade school remains unaddressed by this study. By focusing on relatively severe cases, the study also does not address milder forms of sleep-disordered breathing, which are more prevalent than severe forms and which have been found to increase the risk for behavioral and scholastic problems [4]. Finally, these findings will need to be replicated in completely community-based samples. Children who are referred for clinical evaluation in a sleep clinic are likely to have other problems that brought them to the attention of referring professionals in the first place. Conversely, volunteer controls from the community are often atypical, usually in the direction of better health. These biases complicate comparisons between clinic-referred and community samples.

Of course, such limitations are to be expected in the initial forays into a new research area. But, thanks to Halbower and colleagues, we now have the data needed to justify the more difficult and potentially expensive work to come.

## Abbreviations

MRS: magnetic resonance spectroscopy
OSA: obstructive sleep apnea
